# Low-Dose Radiation Induces Alterations in Fatty Acid and Tyrosine Metabolism in the Mouse Hippocampus: Insights from Integrated Multiomics

**DOI:** 10.1021/acschemneuro.4c00231

**Published:** 2024-08-26

**Authors:** Rekha Koravadi Narasimhamurthy, Babu Santhi Venkidesh, Sampara Vasishta, Manjunath B. Joshi, Bola Sadashiva Satish Rao, Krishna Sharan, Kamalesh Dattaram Mumbrekar

**Affiliations:** † Department of Radiation Biology & Toxicology, Manipal School of Life Sciences, Manipal Academy of Higher Education, Manipal, Karnataka 576104, India; ‡ Department of Ageing Research, Manipal School of Life Sciences, Manipal Academy of Higher Education, Manipal, Karnataka 576104, India; § Directorate of Research, Manipal Academy of Higher Education, Manipal, Karnataka 576104, India; ∥ Department of Radiation Therapy and Oncology, K S Hegde Medical Academy (KSHEMA), Nitte (Deemed to be University), Mangalore, Karnataka 575018, India

**Keywords:** low-dose radiation, neurotoxicity, metabolomics, neurodegeneration, Alzheimer’s disease

## Abstract

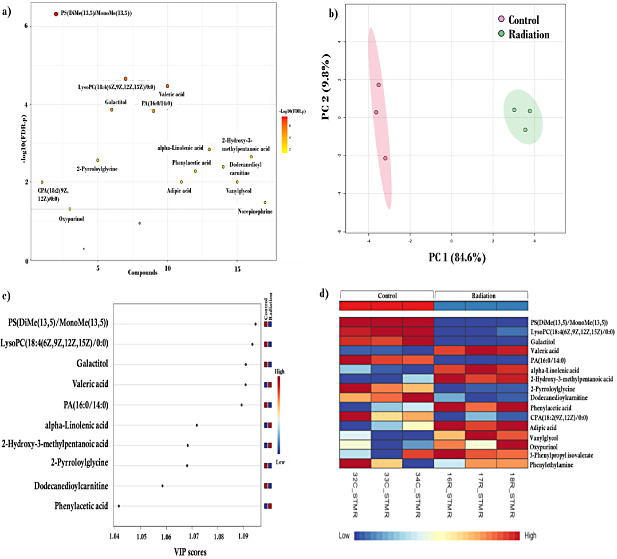

In recent years, there has been a drastic surge in neurological disorders with sporadic cases contributing more than ever to their cause. Radiation exposure through diagnostic or therapeutic routes often results in neurological injuries that may lead to neurodegenerative pathogenesis. However, the underlying mechanisms regulating the neurological impact of exposure to near-low doses of ionizing radiation are not known. In particular, the neurological changes caused by metabolomic reprogramming have not yet been elucidated. Hence, in the present study, C57BL/6 mice were exposed to a single whole-body X-ray dose of 0.5 Gy, and 14 days post-treatment, the hippocampus was subjected to metabolomic analysis. The hippocampus of the irradiated animals showed significant alterations in 15 metabolites, which aligned with altered tyrosine, phenylalanine, and alpha-linolenic acid metabolism and the biosynthesis of unsaturated fatty acids. Furthermore, a multiomics interaction network comprising metabolomics and RNA sequencing data analysis provided insights into gene–metabolite interactions. Tyrosine metabolism was revealed to be the most altered, which was demonstrated by the interaction of several crucial genes and metabolites. The present study revealed the regulation of low-dose radiation-induced neurotoxicity at the metabolomic level and its implications for the pathogenesis of neurological disorders. The present study also provides novel insights into metabolomic pathways altered following near-low-dose IR exposure and its link with neurodegenerative diseases such as Alzheimer’s disease and Parkinson’s disease.

## Introduction

Nervous system disorders are among the leading causes of morbidity and mortality worldwide and contribute vastly to the increasing global burden. Owing to their multifactorial etiology, their various causative mechanisms are largely unexplored. Studies have shown that exposure to environmental toxicants can alter the levels of several metabolites in the brain and can induce neurodegeneration.^1^ Radiation exposure has been linked to the triggering of sporadic cases of neurodegeneration at the early stage of life and manifests as a disease over time.^2^ In the brain, the hippocampus is the site of neurogenesis and controls various complex behavioral capabilities, including cognition, memory, learning, exploratory behavior, and emotions.^3^ Furthermore, it is a vulnerable and plastic region sensitive to various stimuli, and its morphology and cellular and molecular functions are dynamically altered.^4^ Metabolomic perturbations are among the major responses of the hippocampus in the event of neuronal injury.^5^ Moreover, in both postnatal rodents and adults, the subgranular and subventricular zones of the hippocampus harbor neural progenitor cells that are known to be radiosensitive and employed to replenish damaged or dead neural cell populations.^6^

Exposure to ionizing radiation (IR) can occur through natural exposure to background radiation, which can be approximately 3 mGy, and flight travel (approximately 0.01 mGy/h) and space missions (up to 0.5 Gy) or anthropogenic sources such as diagnostic exposure, therapeutic exposure, procedures involving the intake of nuclear medicines, or other sources (1–100 mSv ranging on the part of the body scanned and the radioactive isotope used).^7^ Over the years, the number of individuals undergoing procedures involving IR has increased,^8^ warranting more attention to the short- and long-term consequences of such exposures.^9^ Furthermore, in patients undergoing radiotherapy for brain-related cancers, it has been shown that some portion of surrounding normal tissue can also inadvertently be exposed to lower doses of IR, making it a cause for concern.^10^ Moreover, manned space missions pose risks to the central nervous system (CNS) because of radiation exposure of approximately 0.1–0.5 Gy in the form of highly charged energy particles.^11^ Thus, in these cases, where exposure is unavoidable, there are concerns regarding its long-term implications, particularly in neurodegenerative disorders.^12^ Multiple exposures can lead to accumulated cellular damage that can manifest as cognitive changes, neurological sequelae, or even cancer in some cases.^13^ Additionally, such exposures in children are even more concerning as their brains are still developing and are vulnerable to damage. IR exposure causes impairments in behavior and cognition, changes in neuronal morphology, and neuronal death and induces several alterations at the transcriptomic level.^14^ However, the metabolomic effects of low-dose radiation on the brain have rarely been studied.

High-resolution metabolomic profiling is a recommended hyphenated technique for identifying potential biomarkers involved in the radiation response. It facilitates better translation of radiation-associated pathogenesis postexposure.^15^ Previous studies have investigated the effects of low- and high-dose radiation exposures on metabolomic changes in the brain via low-linear-energy transfer (LET) and high-LET radiation. However, it is well-known that low-dose and high-dose effects induce various metabolite alterations. For example, among low-LET studies, Pazzaglia et al. reported transient biochemical changes in the Raman spectra of the mouse hippocampus at 0.1 Gy, whereas 2 Gy X-ray irradiation induced changes in the TGF-β and DAG/IP3 pathways, also affecting synaptic plasticity and other neuronal functions.^16^ Earlier studies were carried out to understand the metabolomic changes in the extracellular fluid lysate from the brains of glioblastoma patients undergoing radiotherapy^17^ or at higher doses (10 and 30 Gy) in mouse models via the use of serum/plasma samples^18^ or the hippocampus.^19^ One of the earlier studies involving a metabolomics approach following cranial irradiation of 8 Gy revealed several alterations in metabolites involved in the citric acid cycle, neurotransmitter metabolism, and glutamate metabolism in the hippocampus.^20^ Another study investigating the changes in serum profiles after whole-brain irradiation (5 × 2 Gy) reported alterations in the metabolism of branched-chain amino acids such as valine, isoleucine, and leucine.^21^

On the other hand, plasma samples from mice subjected to high-LET whole-body irradiation with 0.5 Gy of ^1^H radiation or ^16^O radiation exhibited alterations in several pathways, such as amino acid metabolism, tyrosine metabolism, lysine metabolism, glycolysis, glycerophospholipid metabolism, and gluconeogenesis.^22^ Wistar rats irradiated with 0.14 Gy of carbon (^12^C) nuclei showed suppressed dopamine turnover in several regions of the brain except in the hippocampus, where the contrast was observed, with decreased levels of norepinephrine in the amygdala.^23^ However, few studies relevant to low-dose radiation, which are representative of typical normal tissue exposure during radiotherapy, diagnostic exposure, or astronaut exposure scenarios, are available. Therefore, it is important to study changes in the brain metabolome and their relevance to neurodegenerative disease pathways. Furthermore, the integrated analysis of transcriptomic and metabolomic data can aid in understanding the multiomics regulation of neurotoxic effects and predict how they can eventually pose a risk in the induction of neurodegeneration. In addition, understanding the metabolites and altered pathways involved will provide insight into the response to low-dose IR.

In the present study, by exploring rodent models, we aimed to understand (a) metabolic differences in the hippocampus in response to 0.5 Gy radiation; (b) gene–metabolite interactions via multiomics approaches involving both RNA sequencing and metabolomics; and (c) pathways specific to neurological functions known to play a role in neurodegenerative disorders.

## Results

### Low-Dose Radiation Induces Metabolic Reprogramming in the Hippocampus

**Figure 1:**
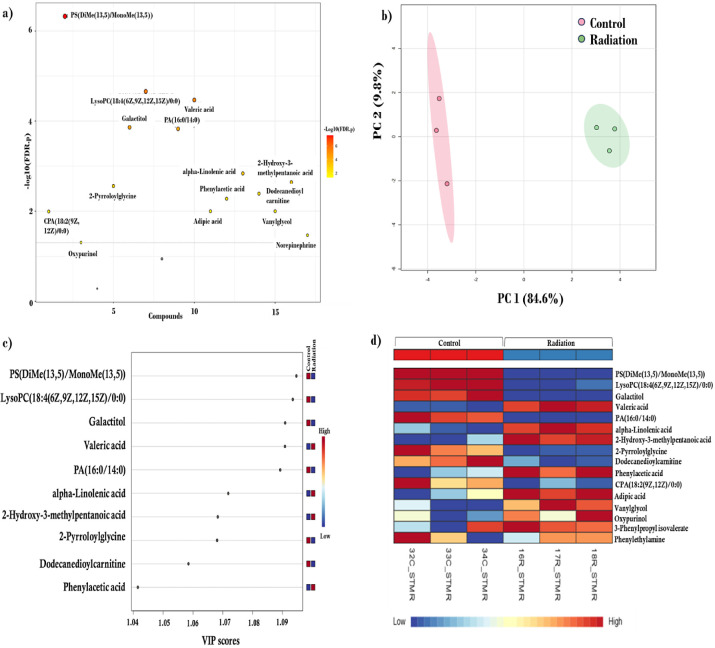
Effects of low-dose radiation exposure on hippocampal metabolites. (a) Number of increased and decreased metabolites. (b) Principal component analysis depicting the variation in the metabolomic data. (c) Variables of importance as a part of the PLS-DA analysis depicting the top ten discriminating metabolites. (d) Fifteen significantly altered metabolites and their levels after low-dose radiation exposure.

A total of 1065 spectral features were obtained, among which 693 features were annotated. Furthermore, after the removal of xenobiotics, 244 metabolites were considered for further statistical analysis via MetaboAnalyst 5.0. After maintaining a stringent parts per million error threshold of 5 ppm, we obtained 17 metabolites, 15 of which were significant (Figure 1a and Table 1). Among the 15 altered metabolites, 7 metabolites were increased, and 8 metabolites were decreased in abundance with respect to the control. Approximately, 33% of the significantly altered metabolites belonged to the fatty acid group, 26% of the significantly altered metabolites belonged to the glycerophospholipid class, and 13% of the metabolites belonged to the carboxylic acid and derivative classes. The remaining metabolites included benzene and substituted derivatives, organo-oxygen compounds, imidazopyrimidines, and phenols.

**Table 1:** List of Significantly Altered Metabolites in the Radiation Group with Respect to the Control Group with Their Log Abundance Values

metabolite	class	log abundance	log_10_(*p*)	*p* value	FDR
phenylacetic acid	benzene and substituted derivatives	4.9348	2.5074	0.0031	0.0053
2-pyrroloylglycine	carboxylic acids and derivatives	3.9994	2.8860	0.0013	0.0028
dodecanedioylcarnitine	carboxylic acids and derivatives	3.1163	2.6652	0.0022	0.0041
caleric acid	fatty acids	5.2594	5.2214	0.0000	0.0000
alpha-linolenic acid	fatty acids	4.3284	3.2901	0.0005	0.0015
2-hydroxy-3-methylpentanoic acid	fatty acids	4.4815	3.0281	0.0009	0.0023
adipic acid	fatty acids	4.5494	2.1507	0.0071	0.0100
CPA(18:2(9Z,12Z)/0:0)	glycerophospholipids	4.4420	2.1101	0.0078	0.0101
PS(DiMe(13,5)/MonoMe(13,5))	glycerophospholipids	0.0000	7.5563	0.0000	0.0000
lysoPC(18:4(6Z,9Z,12Z,15Z)/0:0)	glycerophospholipids	2.5920	5.5885	0.0000	0.0000
PA(16:0/14:0)	glycerophospholipids	0.0000	4.3598	0.0000	0.0001
oxypurinol	imidazopyrimidines	4.5739	1.3603	0.0436	0.0494
galactitol	organooxygen compounds	4.4791	4.4908	0.0000	0.0001
vanylglycol	phenols	5.5440	2.1699	0.0068	0.0100
norepinephrine	phenols	4.1899	1.5519	0.0281	0.0341

### PCA and PLS-DA Revealed Distinct Clusters between Groups

**Figure 2:**
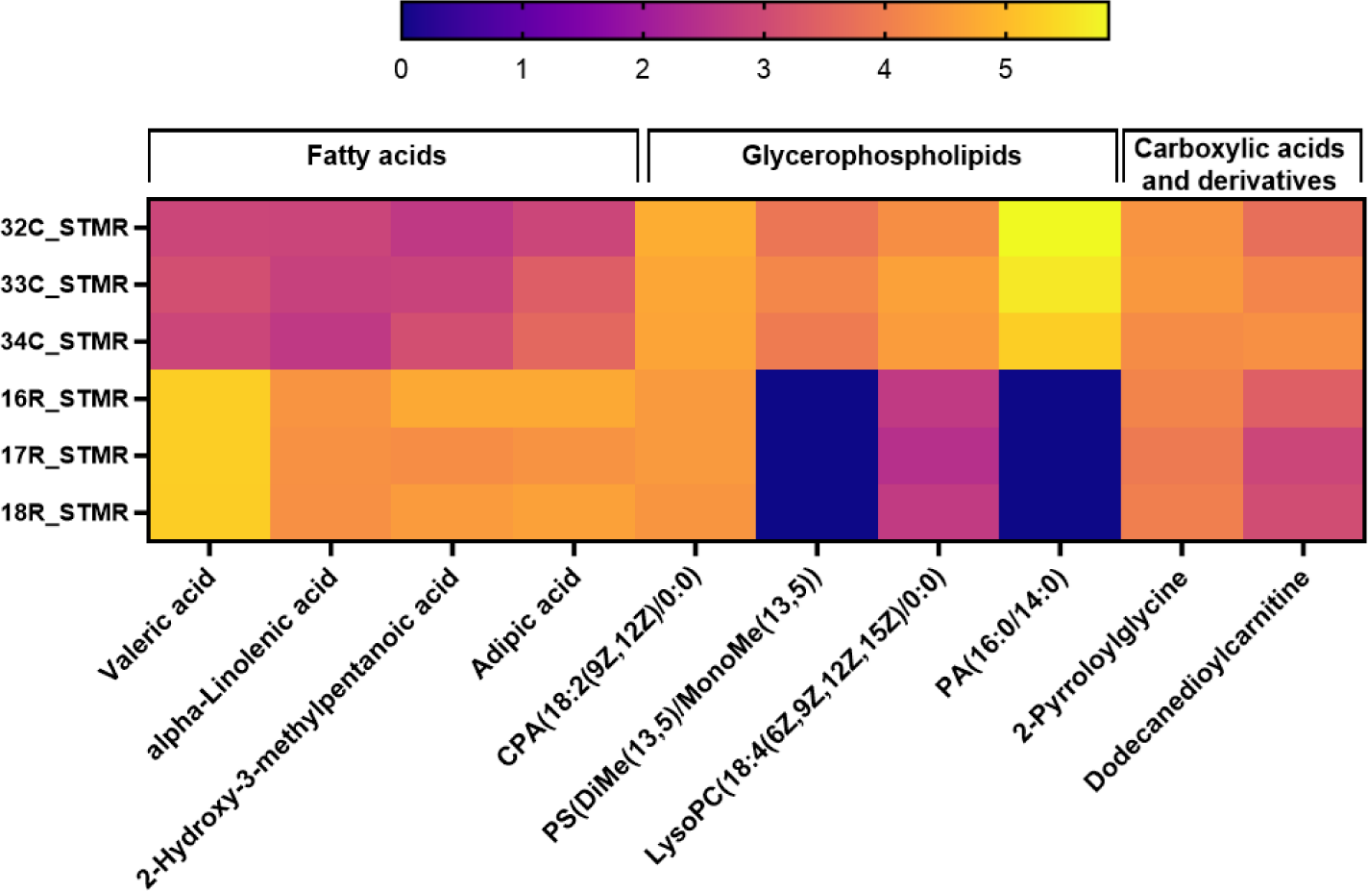
Effects of low-dose radiation exposure on hippocampal metabolite classes. Heatmap representing the levels of different metabolites belonging to fatty acids, glycerophospholipids, and carboxylic acids and derivatives between groups.

**Figure 3:**
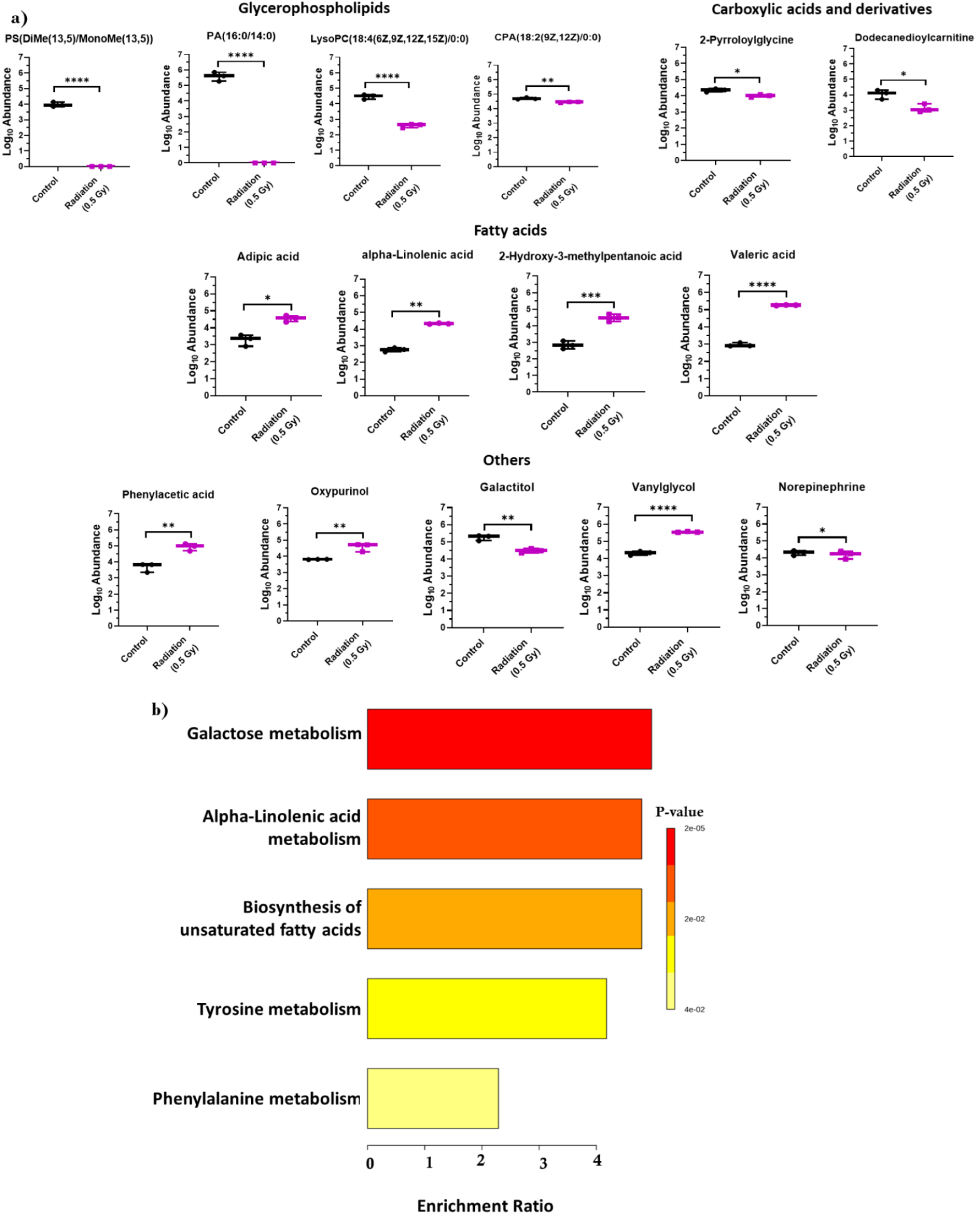
Effects of low-dose radiation exposure on hippocampal metabolites and pathways. (a) Box-whisker plots of metabolites upregulated and downregulated in radiation (*n* = 3, *p < 0.05, **p < 0.01, ***p < 0.001, ****p < 0.0001). (b) Different metabolomic pathways enriched by significantly altered metabolites after radiation exposure.

Principal component analysis revealed that metabolomic metabolites displayed maximum variation between the groups, with components one and two displaying 84.6% and 9.8% variation, respectively (Figure 1b), which prompted us to perform further focused analysis. To derive the most variable metabolites contributing to a maximum distinction between the two groups, we carried out a PLS-DA analysis. The top 5 discriminating metabolites according to their VIP scores between the control and radiation groups were PS(DiMe(13,5)/MonoMe(13,5)), lysoPC(18:4(6Z,9Z,12Z,15Z)/0:0), galactitol, valeric acid, and PA(16:0/14:0) (Figure 1c). A cluster heatmap depicted PS(DiMe(13,5)/MonoMe(13,5)), lysoPC(18:4(6Z,9Z,12Z,15Z)/0:0), galactitol, PA(16:0/14:0), and 2-pyrroloylglycine as the five metabolites with the greatest decreases, whereas valeric acid, alpha-linolenic acid, 2-hydroxy-3-methylpentanoic acid, phenylacetic acid, and adipic acid were the five metabolites with the greatest increases following radiation (Figure 1d). The metabolite levels of fatty acids, glycerophospholipids, and carboxylic acids and their derivatives are represented in a heatmap (Figure 2). The levels of several metabolites, such as valeric acid, alpha-linolenic acid, phenylacetic acid, and lysoPC(18:4(6Z, 9Z, 12Z, 15Z)/0:0), altered in the radiation group are represented in the form of a box plot (Figure 3a).

### Pathway Enrichment Analysis Revealed That Low-Dose Radiation Modulates Amino Acid and Fatty Acid Metabolism

To understand the role of altered metabolites in different pathways, we carried out a pathway enrichment analysis via the KEGG database. Among the top five pathways that were altered after radiation exposure, two fatty acid-related pathways, namely, alpha-linolenic acid metabolism and biosynthesis of unsaturated fatty acids, were enriched, whereas two amino acid metabolism pathways, namely, tyrosine metabolism and phenylalanine metabolism, were altered (Figure 3b). Furthermore, we performed a joint pathway analysis that revealed that tyrosine metabolism was the most altered metabolic pathway.

### Metabolomic and Transcriptomic Integrated Analysis Identifies Hub Genes Involved in the Regulation of Tyrosine Metabolism

To understand the association between transcriptomic regulation and metabolite levels, we performed multiomics analysis by integrating the list of significant metabolites obtained in the study with the set of differentially expressed genes (DEGs) between the same two groups of animals obtained from a set of experimental hippocampal transcriptome studies via the online tool Metabridge. We found that the dopamine beta-hydroxylase (*Dbh)* gene from our list of differentially expressed genes was correlated with the metabolite norepinephrine.

**Figure 4:**
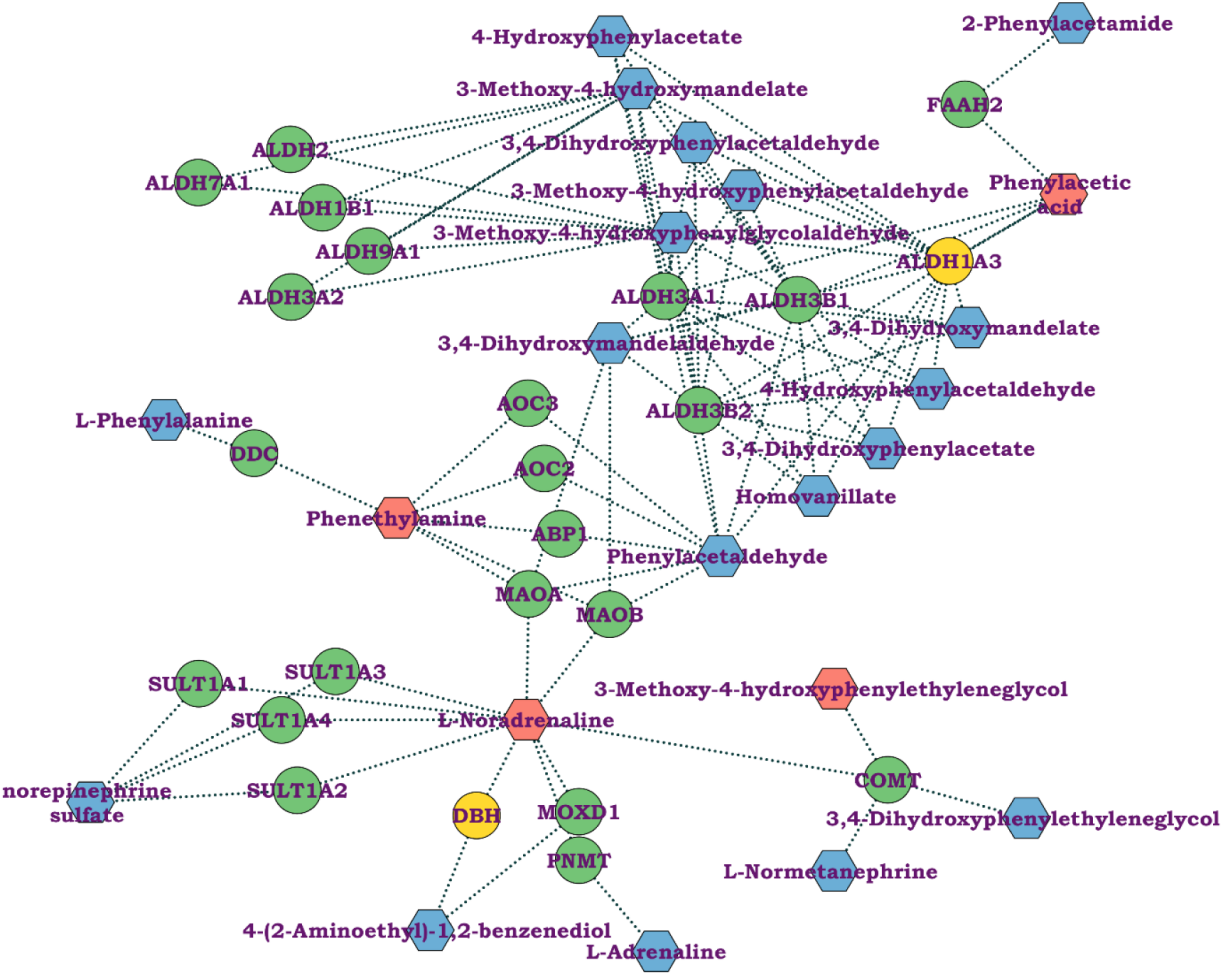
Effects of low-dose radiation exposure on amino acid metabolism. Network analysis shows the nodes connecting tyrosine metabolism and its interacting metabolites and genes. Genes in yellow circles indicate genes from our transcriptomic data, genes in green circles depict the hub genes involved in pathway regulation, those in pink hexagons indicate metabolites altered in our study, and those in blue hexagons indicate hub metabolites involved in pathway regulation.

Furthermore, integrated transcriptomic and metabolomic analyses via MetScape revealed that tyrosine metabolism was the most strongly affected pathway. We further performed a network analysis of the interactions of metabolites altered in the radiation group with the set of DEGs from our previous study along with their hub genes and metabolites (Figure 4). We found that the metabolites phenylacetic acid and noradrenaline, along with two other altered metabolites that remained insignificant, namely, 3-methoxy-4-hydroxyphenylethylene glycol and phenethylamine were involved in tyrosine metabolism regulation. Furthermore, two genes from our list, namely, aldehyde dehydrogenase 1 family member A3 (*Aldh1a3)* and *Dbh,* were also involved in pathway regulation. Here, *Aldh1a3*, along with other interacting genes, has also been reported to regulate the metabolites of the amino acid pathway.

## Discussion

Low-dose radiation exposure can hamper several cellular processes and pathways by interfering with the expression of genes, proteins, and, ultimately, metabolites. Although there are many conventional methods for assessing the radiation dose–response, obtaining quick and precise biomarkers and understanding the correlation between gene expression and the level of metabolites are needed. Exposures of up to 0.5 Gy of radiation are a probable scenario in manned space missions, which have increased in frequency over the years.^11^ Furthermore, in cases of radiotherapy, there are instances of normal tissue exposure up to several milli grays of radiation with evidence that the hippocampus becomes inadvertently exposed to approximately 155 mGy per fraction. In such cases, if we consider fractionated exposures, the cumulative dose can reach 1 Gy or more, which would then be a high dose.^10^ Therefore, there is a need to understand the mechanisms at play when an individual is exposed to such doses. The present study employed an untargeted metabolomics approach to scan alterations in various metabolites in the hippocampus in response to near-low-dose radiation exposure to understand the further implications of these alterations and the pathways affected. Our findings suggest that near-low-dose radiation can primarily alter fatty acid and amino acid levels and alter closely linked pathways, such as those involved in neurodegenerative pathogenesis.

In the present study, we detected a drastic decrease or complete absence in the levels of four glycerophospholipids, namely, PS(DiMe(13,5)/MonoMe(13,5)), lysoPC(18:4(6Z,9Z,12Z,15Z)/0:0), CPA(18:2(9Z,12Z)/0:0), and PA(16:0/14:0). PLS-DA further revealed that three of these four metabolites were among the top 5 discriminators of the group, with PS(DiMe(13,5)/MonoMe(13,5)) and lysoPC(18:4(6Z,9Z,12Z,15Z)/0:0) being the top two variable metabolites. Phospholipids play crucial roles in maintaining cell structure and membrane integrity, regulating cellular signaling, contributing to neurogenesis and plasticity, and overall enhancing brain health.^24^ The decrease in the levels of these metabolites could be due to overdrive in signaling processes, particularly those activated as a result of the radiation-induced damage response, leading to increased usage of these signaling molecules or indicating membrane damage. These findings are consistent with our *in vivo* findings, where we reported neuronal damage in the hippocampus.^14^ Furthermore, a decrease in the level of phospholipids is correlated with the inflammatory response,^25^ which was again observed through the activation of astroglia in the hippocampus after exposure to 0.5 Gy radiation.^14^ Previously, a dose-dependent decrease in the levels of phosphatidylcholines was reported in the serum of head and neck cancer patients who underwent radiotherapy.^25^ Furthermore, several studies have reported alterations in the levels of phospholipids associated with neurological disorders,^26^ with phospholipidomics being identified as a crucial technique for identifying its role in neurological disorders and thus serving as an important biomarker.^27^

We also revealed that the levels of metabolites belonging to the fatty acid group, such as 3-phenylpropyl isovalerate, 2-hydroxy-3-methylpentanoic acid, valeric acid, and adipic acid, significantly increased after radiation exposure. Like phospholipids, fatty acids also serve as signaling molecules by modulating several ion channels, receptors, proteins, and enzymes; constitute the composition of cell membranes; and serve as sources of energy for neurons. However, they are also imperative in immunomodulation, as they can activate many inflammatory molecules, such as Toll-like receptors and cytokines.^28^ Since we observed a neuroinflammatory response in our parallel study,^14^ we hypothesized that this neuroinflammatory response could have resulted from lipid and fatty acid metabolomic reprogramming in the mouse hippocampus as a result of low-dose radiation exposure. Valeric acid, whose level was increased in our study, is produced by the gut microbiota and has been shown to play a role in neuroinflammation and worsened neurological outcomes.^29^ Since our experimental setup involved the administration of whole-body radiation, this could have altered the gut microbiota metabolites, which have previously been postulated to induce changes in the brain via the gut–brain axis.^30^ Alpha-linolenic acid, another fatty acid whose level is increased in the hippocampus, promotes neurogenesis and synaptogenesis, serves as an anti-inflammatory and radical scavenging agent, and can also help sustain the integrity of the blood–brain barrier.^31,32^ This could also be a modulator of the antioxidant enzyme activation that we observed in our parallel study.^14^ All of the altered glycerophospholipid and fatty acid levels contributed to the alteration of alpha-linolenic acid metabolism and the biosynthesis of unsaturated fatty acids when we performed pathway enrichment analysis. Previous studies have shown that lipids and fatty acids are implicated in neurodegenerative pathogenesis and hence proved to be useful targets for therapy.^33^

Radiation exposure results in a reduction in the levels of norepinephrine, also known as noradrenaline, which plays a crucial role as a neuromodulator influencing the activity of both neurons and non-neuronal cells, such as microglia and astrocytes.^34^ More importantly, it is also a neurotransmitter and is mainly responsible for functions such as wakefulness, behavior and memory, arousal, and alertness.^35^ However, the greatest concern among its activities is that the neurons developed from the influence of norepinephrine in the locus coeruleus that project into the rest of the brain are said to be the early targets of various neurodegenerative disorders.^35^ Our pathway enrichment analysis revealed that one of the pathways that was modulated was the tyrosine metabolism pathway. Elevated tyrosine metabolism is a trigger for the production of norepinephrine through its release into the synaptic cleft from the presynaptic terminal.^36^ Hence, the alteration observed in the tyrosine pathway could be one of the reasons for the changes in the level of norepinephrine. This could also explain the increased neuronal pyknosis, decreased number of mature neurons, and increased neuroinflammation through reactive astrogliosis that we observed in a parallel study involving similar experimental exposure.^14^ In addition to tyrosine metabolism, norepinephrine also plays a role in the synaptic vesicle cycle, as revealed by pathway enrichment.

As stated before, pathway enrichment of the significantly affected metabolites revealed alterations in tyrosine and phenylalanine metabolisms. These amino acid synthesis pathways are involved in the biosynthesis of neurotransmitters and pathways associated with diseases such as Alzheimer’s disease and Parkinson’s disease. Tyrosine metabolism directly regulates the synthesis of l-DOPA to dopamine and indirectly regulates the synthesis of norepinephrine,^37^ whereas alterations in phenylalanine can cause changes in the levels of catecholamine neurotransmitters.^38^ Notably, abnormal amino acid ratios and metabolism have been reported in the early stages of Alzheimer’s disease,^39^ indicating that alterations in these metabolites can have serious neurodegenerative implications. To date, studies reporting altered amino acid metabolism in the brain due to low-dose radiation are very rare. However, Yamaguchi et al. reported changes in a few amino acid residues in the serum and sequences of individuals exposed to low-dose radiation.^40^ Another study by Fónagy et al. also showed that upon a single whole-body dose of 0.5 Gy neutrons on embryonic day 17, approximately 40% of newborn mice died, and the brain weight decreased in approximately 30–35% of the progeny. Protein synthesis was also found to decrease *in utero*, as indicated by the 40% decrease in the incorporation of labeled amino acids in histone and nonhistone proteins, as well as reduced aminoacyl-tRNA in the brain.^41^ Therefore, given the connection between low-dose radiation and neurotoxicity as well as the demonstrated involvement of these metabolites and pathways in cases of neurotoxicity contributing to neurodegeneration, it can be inferred that low-dose radiation might trigger neurotoxicity by perturbing various amino acid metabolic pathways. Furthermore, since manned space missions involve exposure to radiation doses of up to 0.5 Gy from high charge and energy (HZE) nuclei, elucidating the metabolomic changes can help determine the risks to the brain associated with such missions.^11^

The connectivity between genes and metabolites is a cyclic process that initiates with genes encoding a particular protein that, in turn, degrades into metabolites or uses metabolites for post-translational modifications and cell signaling processes that again assemble the same set of genes and proteins. Therefore, it is crucial to study the regulation of metabolite levels in response to various external factors because of their active participation in different cellular processes. Numerous studies have successfully employed metabolomics to obtain detailed insights into the mechanisms prevailing in neurodegenerative disorders.^42^ However, a complete assessment of the entire spectrum of changes cannot be achieved via only a single omics approach, making it essential to employ multiple platforms to identify the most comprehensive set of potential biomarkers and their interactions.^43^ Recently, the use of a multiomics approach for the comprehensive analysis of transcriptomic, proteomic, and metabolomic changes triggered after exposure to xenobiotics has taken precedence. Integrated multiomics analysis helps identify converging pathways and the crosstalk among different genes, enzymes, and metabolites. Two such genes are *Aldh1a3,* which is a prominent stem cell marker involved in mesenchymal differentiation and is known mainly for its role in the progression of glioblastoma,^44^ and *Dbh*, which is involved in the synthesis and conversion of norepinephrine into different neurons and cell phenotypes, whose decrease in the brain could be one of the indicators of neurological disorders.^45^ Both of these genes were found to modulate tyrosine metabolism along with some of the metabolites found to be altered in the hippocampus. A previous study employing integrated metabolomics-DNA methylation analysis revealed an increase in total amino acid synthesis.^19^ Another study involving joint transcriptomic and metabolomic analysis revealed alterations in nucleotide, amino acid, carbohydrate, lipid, and fatty acid metabolism, thus emphasizing the utility of multiomics approaches.^46^

The findings of the present study provide insight into the metabolomic response to near-low-dose radiation exposure, which is characterized by alterations in amino acid metabolism pathways, such as tyrosine and phenylalanine metabolism and fatty acid biosynthesis and metabolism, which play crucial roles in inducing neurotoxicity. Our study enhances the understanding of the link between their exposure and the manifestation of neurotoxicity in neurodegenerative-like conditions through a metabolomic approach. We further employed integrated transcriptomic and metabolomic analyses to provide a holistic outlook and to improve our understanding of the pathogenetic mechanism underlying the damage associated with near-low-dose exposure to radiation. The dose employed in this study provides an understanding of the primary mechanisms at play during isolated-dose exposure, helps establish baseline data on the effects of low-dose radiation on the hippocampus, and allows us to consider repeated exposure settings. Furthermore, this dose may help us set a threshold dose post which there might be a dramatic change in the cellular responses, which could be more damaging in nature, whereas 0.5 Gy may be studied for its possible therapeutic effects (if any) or lie within the range where damage repair is still possible or probable. Furthermore, the findings of this study could be compared with the findings of studies employing fractionated doses of up to 0.5 Gy and highlight the similarities and dissimilarities between the mechanisms involved in singular and cumulative effects at the same dose. This approach would also help correlate the metabolomic changes observed in our study with other histological or molecular changes observed in studies employing 0.5 Gy. Additionally, singular exposure allows for the observation of acute effects postexposure. This will further our knowledge of the mechanistic alterations and help predict responses at higher doses on the basis of the activated pathways identified. However, it would further benefit us if we profile the metabolomic signatures at even lower doses and in repeated exposure scenarios, which will aid in gathering more insight into responses at doses closer to diagnostic exposures. Furthermore, screening of distinct metabolites in large populations can aid in identifying biomarkers of neurotoxicity associated with future exposure. Moreover, identifying unique metabolites can provide useful therapeutic targets for their activation or inhibition and subsequent amelioration of near-low-dose radiation exposure-induced neurotoxicity.

## Materials and Methods

### Animal Maintenance and Exposure

C57BL/6 mice (approximately 5 weeks old) were maintained in the Central Animal Research Facility after ethical approval was obtained from the Institutional Animal Ethics Committee, Manipal Academy of Higher Education, Manipal (IAEC/KMC/108/2019). The animals were maintained in a controlled environment with a temperature of 20 °C ± 2 °C and a 12-h light and dark cycle. Six animals in two groups, namely, the control and radiation groups, were used for the study. The radiotherapy facility at the Shirdi Sai Baba Cancer Hospital, Manipal, was used for animal irradiation. The animals were irradiated by placing them in special restrainers made of acrylic sheets. Before irradiation, a dose delivery simulation was conducted on the restrainer via MONACO TPS 5.11 (Elekta, Sweden), which employs an isocentric technique to ensure precise irradiation of the area of interest in the field. Accordingly, the initial gantry, collimator, and couch angles were also set to maintain alignment. The animals in the radiation group were subjected to X-ray through two beams, 0.25 Gy from the antero-posterior direction and 0.25 Gy from the postero-anterior direction, to derive a cumulative total dose of 0.5 Gy of whole-body single-dose radiation via a Versa-HD Linear Accelerator (Elekta, Sweden) via 6 MV photon beams in an antero-posterior–postero-anterior (AP–PA) parallel opposed field arrangement via the source-to-axis distance (SAD) technique, while the control animals were sham irradiated. The dose rate of the irradiator was >12 Gy/h, and the irradiator typically delivered approximately 3–6 Gy/min at Dmax. Fourteen days after treatment, the animals were euthanized by cervical dislocation, and the hippocampus was isolated and stored at −80 °C until further processing.

### Sample Preparation for Mass Spectrometry

Sample preparation was performed according to a previously described protocol with slight modifications.^47^ Hippocampal tissues (from three animals from each group) were weighed and minced in methanol and water (4:1, v/v) at a final concentration of 20 mg/mL. The samples were then briefly vortexed for 30 s, snap-frozen in liquid nitrogen, and sonicated for 5 min in 3 cycles. The samples were incubated at −20 °C for 1 h, followed by centrifugation at 12000 rpm for 10 min at 4 °C. The supernatant was then collected and lyophilized, and the residue was resuspended in 100 μL of cold acetonitrile and water (50:50, v/v) containing 1% formic acid. The samples were further centrifuged at 12000 rpm for 10 min at 4 °C, and the supernatant was collected and stored at −80 °C until LC–MS injection.

### LC/MS Conditions

Mass spectrometry was carried out via an ESI-QTOF instrument (Agilent 6250 TOF-MS, Agilent Technologies, USA) coupled with a high-performance liquid chromatography system (Agilent 1200 series, USA). The sample was centrifuged, and the pellet was reconstituted in 100 μL of water:acetonitrile (95:5) containing 0.1% formic acid. The analysis of the samples was performed through ESI in positive mode in triplicate with an injection volume of 5 μL and a run time of 45 min. The metabolites were eluted via an Agilent analytical column (ZORBAX Eclipse XDB C18, 4.4 × 250 mm, 5 μm). Mobile phase A consisted of water with 0.1% formic acid, and mobile phase B consisted of 90% acetonitrile with 0.1% formic acid. The conditions of the mass spectrometry run were as follows: a gas temperature of 250 °C, a gas flow of 8 L/min, a nebulizer pressure of 40 psig, and an ESI capillary voltage of 3500 V.

### Metabolomic Data Analysis

The raw data obtained in .d format were converted to mzML format via MS Convert. The mzML converted files were then processed via MetaboAnalyst 5.0,^48^ where the sample data were checked for quality and integrity. Further processing and annotation were carried out in positive mode using a tolerance limit of ±5 ppm. The HMDB was used to identify the metabolites, and the raw data and statistical analysis were carried out via MetaboAnalyst 5.0. The data were then normalized, log-transformed, and autoscaled. Fold change analysis and *t* tests were carried out to identify the differences among the metabolites. A *p* value of <0.05 was considered to indicate statistical significance. Principal component analysis (PCA) of the metabolites was carried out to visualize the variability between the data sets of the different groups. Partial least-squares discriminant analysis (PLS-DA), a statistical tool that determines and classifies the metabolites that contribute the most to the separation between two groups, was also carried out. This was accomplished by attributing a variable of importance (VIP) metric score to the metabolites on the basis of the metabolite intensities. Furthermore, enrichment analysis was performed to understand the metabolite–pathway–disease associations via the KEGG database via MetaboAnalyst 5.0.

### Metabolite–Gene Interaction Analysis

The HMDB IDs of the selected metabolites were input into the online tool Metabridge,^49^ and a list of genes regulating the metabolite/enzyme levels was obtained. The list of genes was compared with our published hippocampal transcriptomic data^14^ to identify commonly altered genes. Additionally, we used Metscape,^50^ a plugin in Cytoscape,^51^ to find a direct correlation between the DEGs identified in our previous study and the list of metabolites identified in the current study and the pathways in which they are involved.

## Supporting Information Available

List of metabolites and their respective details, including RT, theoretical mass, *m*/*z*, chemical formula, adducts, delta (ppm), HMDB ID, and KEGG ID (PDF)

## Notes

Animals were obtained from the Central Animal Research Facility, Kasturba Medical College, Manipal Academy of Higher Education, Manipal, after receiving ethical approval from the Institutional Animal Ethics Committee (IAEC/KMC/108/2019), Manipal Academy of Higher Education, Manipal.
