# Supplementary material for: Low-Dose Radiation Induces Alterations in Fatty Acid and Tyrosine Metabolism in the Mouse Hippocampus: Insights from Integrated Multiomics
Source: ACS Chem Neurosci. 2024 Aug 26;15(18):3311–20. doi: 10.1021/acschemneuro.4c00231 (PMC11413841; doi:10.1021/acschemneuro.4c00231)
Supplement: Supplementary file 1 — cn4c00231_si_001.pdf [file cn4c00231_si_001.pdf]

Supporting Information

Low-dose Radiation Induces Alterations in Fatty Acid and Tyrosine Metabolism in the Mouse Hippocampus: Insights from Integrated Multiomics

Rekha Koravadi Narasimhamurthy[1], Babu Santhi Venkidesh [1], Sampara Vasishta[2], Manjunath B Joshi[2], Bola Sadashiva Satish Rao[1,3], Krishna Sharan[4], Kamalesh Dattaram Mumbrekar[1*]

[1]Department of Radiation Biology & Toxicology, Manipal School of Life Sciences, Manipal Academy of Higher Education, Manipal, 576104, Karnataka, India

[2]Department of Ageing Research, Manipal School of Life Sciences, Manipal Academy of Higher Education, Manipal, 576104, Karnataka, India

[3]Directorate of Research, Manipal Academy of Higher Education, Manipal, 576104, Karnataka, India

[4]Department of Radiation Therapy and Oncology, K S Hegde Medical Academy (KSHEMA), Nitte (Deemed to be University), Mangalore, 575018, Karnataka, India

*Corresponding author
Dr. Kamalesh Dattaram Mumbrekar
Associate Professor
Department of Radiation Biology & Toxicology
Manipal School of Life Sciences
Manipal Academy of Higher Education
Manipal-576104, India
Email: kamalesh.m@manipal.edu
Ph No: 9880100901

Table S1: List of metabolites and the respective details including RT, theoretical mass, m/z, chemical formula, adducts, delta (ppm), HMDB ID and KEGG ID.

Metabolite	Class	RT (sec)	Theoretical mass	m/z	Formula	Adducts	Delta (ppm)	HMDB ID	KEGG ID
CPA(18:2(9Z,12Z)/0:0)	Glycerophospholipids	1079.6370	416.2328	417.2401	$C_{21}H_{37}O_6P$	M+H	0	HMDB0007007	NA
PS(DiMe(13,5)/MonoMe(13,5))	Glycerophospholipids	313.3407	965.6357	966.6434	$C_{53}H_{92}NO_{12}P$	M+H	0	HMDB0061577	NA
Oxypurinol	Imidazopyrimidines	286.1936	152.0334	153.0405	$C_5H_4N_4O_2$	M+H	1	HMDB0000786	C07599
Phenylethylamine	Benzene and substituted derivatives	410.1121	121.0891	122.0965	$C_8H_{11}N$	M+H	1	HMDB00012275	C05332
Norepinephrine	Phenols	775.5023	169.0739	170.0814	$C_8H_{11}NO_3$	M+H	1	HMDB0000216	C00547
2-Pyrroloylglycine	Carboxylic acids and derivatives	300.2302	168.0535	169.0610	$C_7H_8N_2O_3$	M+H	2	HMDB0059778	NA
Galactitol	Organooxygen compounds	439.1129	182.079	183.0869	$C_6H_{14}O_6$	M+H	3	HMDB0000107	C01697
LysoPC(18:4(6Z,9Z,12Z,15Z)/0:0)	Glycerophospholipids	904.3183	515.3012	516.3102	$C_{26}H_{46}NO_7P$	M+H	3	HMDB0010389	NA
3-Phenylpropyl isovalerate	Fatty acids	1409.3834	220.1463	221.1528	$C_{14}H_{20}O_2$	M+H	4	HMDB0036389	NA
PA(16:0/14:0)	Glycerophospholipids	1925.9873	620.4417	621.4512	$C_{33}H_{65}O_8P$	M+H	4	HMDB0114833	NA
Valeric acid	Fatty acids	1826.5483	102.0681	103.0758	$C_5H_{10}O_2$	M+H	4	HMDB0000892	C00803
Adipic acid	Fatty acids	835.2085	146.0579	147.0645	$C_6H_{10}O_4$	M+H	4	HMDB0000448	C06104
Phenylacetic acid	Benzene and substituted derivatives	1271.7601	136.0524	137.0603	$C_8H_8O_2$	M+H	4	HMDB0000209	C07086
alpha-Linolenic acid	Fatty acids	1794.1275	278.2246	261.2204	$C_{18}H_{30}O_2$	M+H-H2O	5	HMDB0001388	C06427

Dodecanedioylcarnitine	Carboxylic acids and derivatives	1605.5250	373.2464	374.2554	$C_{19}H_{35}NO_6$	M+H	5	HMDB0013327	C03299
Vanylglycol	Phenols	2046.5602	184.0736	185.0818	$C_9H_{12}O_4$	M+H	5	HMDB0001490	C05594
2-Hydroxy-3-methylpentanoic acid	Fatty acids	784.8957	132.0786	133.0866	$C_6H_{12}O_3$	M+H	5	HMDB0000317	NA
